# Facility evaluation of resigned hospital physicians:managerial implications for hospital physician manpower

**DOI:** 10.7603/s40681-016-0023-3

**Published:** 2016-11-17

**Authors:** Kao-Chi Cheng, Tsung-Lin Lee, Yen-Ju Lin, Chiu-Shong Liu, Cheng-Chieh Lin, Shih-Wei Lai

**Affiliations:** 1 College of Medicine, China Medical University, No. 2, Yuh-Der Road, 404 Taichung, Taiwan; 2 Department of Family Medicine, China Medical University Hospital, 404 Taichung, Taiwan; 3 Department of Family Medicine, Show Chwan Memorial Hospital, 500 Changhua, Taiwan

**Keywords:** Physician who left hospital, Retention, loyalty, Job satisfaction

## Abstract

**Introduction:**

Turnover of physicians might be responsible for reducing patients’ trust and affecting hospital performance. This study aimed to understand physicians’ psychological status regarding their hospital work environment and the resources of independent practitioners.

**Method:**

This was a cross-sectional study with 774 physicians who had resigned from hospitals and were now practicing privately in clinics in Taichung City as its study population. A mail survey with a multidimensional questionnaire was sent to each subject.

**Results:**

This study revealed that older physicians were less satisfied regarding the work environment in their respective former hospitals. Male physicians were found to be more satisfied with the tangible resources of their hospitals. Internal medicine physicians were found to be less satisfied overall with the intangible resources. Gynecologists and pediatricians were found to be more satisfied with their hospital environments. The physicians who worked long hours per week reported that they were less satisfied with their job content. The physicians who had opportunities to learn advanced skills and enhance their knowledge were more satisfied with their hospital environment, tangible resources, and intangible resources. In addition, physicians in private hospitals were found to be more satisfied with their job content, but they were less satisfied with work motivation and retention and intangible resources. In addition, physicians who worked in hospitals located in Taichung city reported that they were less satisfied with their tangible resources than the physicians working in hospitals outside of the city.

**Conclusion:**

This study focused on the satisfaction of physicians who had already left their respective hospitals instead of current retained physicians. From this study, it is our recommendation that hospital managers should pay closer attention to the real needs and expectations of the physicians they employ, and managers should consider adjusting their managerial perspectives when establishing new human resources policies or making decisions.

## 1. Introduction

Over the past 50 years, organizational behavior scholars have attempted to understand how the issue of loyalty relates to the retention of employees by evaluating their levels of job satisfaction [1-4]. High employee retention is the key to service excellence and operational excellence [5]. It stands to reason that if employers treat their employees as valued contributors, then the employees will stay and be satisfied with their jobs. To this end, companies have trained their managers to offer competitive compensation plans with increasing benefits to secure their employees’ loyalty and retention. Despite such efforts, many health care organizations are experiencing a shortage of employees and high turnover rates [6]. A working culture that fosters high employee motivation is necessary for an organization to compete in the highly dynamic and competitive environments of today’s society. Managers need to implement effective human resource strategies and policies to establish and maintain such a working culture in any organization. High employee turnover rates can have a significantly negative impact on operation results for managers and organizations [7]. When an employee is planning to resign, productivity and quality of work is likely to decline. Meanwhile, improving employee retention can result in positive outcomes for an organization, including workforce stability, employee selection cost savings, and managers having to spend less time interviewing prospective employees and integrating replacements into the work system [7].

The importance of relational factors in explaining turnover is evident in the context of non-physicians [8-11]. Favorable perceptions of global work satisfaction, autonomy in the workplace, professional status, teaching activities, clinical resources and activities, professional relationships, and institutional governance all correlate inversely with intentions of leaving [12]. Many health care researchers and administrators have noted either the importance of job satisfaction on a variety of organizational variables or on personal variables [13]. In addition, competing demands between professional and personal roles often result in conflict for employees [14]. Some studies indicate the cost of turnover can be 1.5 times of an employee’s annual salary [15]. Furthermore, when employees leave, their duties are shifted to the remaining personnel who subsequently are made to feel obligated to shoulder the additional burden [16]. Generally speaking, many determinants, including lowered loyalty to institutions, loss of balance between work and family, and organizational or personal variables will cause a high turnover rate and lower job satisfaction. [17, 18]

Compared with other medical experts, physicians play an important and professional role in the medical teams of hospitals. The turnover of physicians threatens the continuity of care for patients and is a significant expense for health care organizations [19]. Although physician turnover in a health care organization can incur substantial costs, little formal attention has thus far been given to estimating or modeling the financial impact of such turnover on revenues [20]. The cost of physician recruitment and adverse consequences of turnover have led to significant concerns among all administrators of health care organizations. For example, the cost of physician recruitment can range from $236,383 USD for family medicine physicians to $264,645 USD for pediatricians, and even recruiting a new primary care physician who is emerging from a training program costs approximately $236,000 USD [21]. Beasley, Karsh, Hagenauer, March-and, & Sainfort also found that replacing a physician costs about $250,000 USD [22]. In Taiwan, the cost of hiring a new doctor may be less than it is in America, but it is still higher than retaining a current physician [23]. All leaders or managers in health care organizations have attempted to keep costs down to retain physicians and to also decrease the turnover rate of their physicians.

Job satisfaction is an important determinant of physician retention and turnover, and may also affect performance [24, 25]. Mick has also argued that physician turnover might reduce the trust patients have in providers and health care organizations [26]. Successful health care organizations emphasize attracting human resource assets and aggressively seek to prevent or resolve high employee turnover. Collins & Collins have pointed out that understanding the key components surrounding the importance of measuring employee turnover, learning how it affects patient care, and realizing what is needed to retain quality employees is central to the resolution of physician turnover [16]. They suggested that organizations should focus on the following issues in order to maintain their qualified workforce in the long term: communication; decision-making; compensation, benefits, and career development; recruitment; appreciation and understanding; and management.

In recent decades, more attention has been paid to the idea that social relationships at work may influence a physicians’ job satisfaction and their decision whether or not to withdraw from practice [27, 28]. As physicians’ practice and daily work routines are based on interactions with colleagues, staff, and patients, the quality of the relationships with members of each group may assume critical importance in physicians’ decisions to continue working with or withdrawing from their practice settings [13]. The importance of workplace relationships for physicians can also be related to what Portes has pointed out-the two types of motivation for workplace social relationships: instrumental motivation and consummatory motivation [29]. It has been suggested that physicians might build positive relationships with colleagues, staff, and patients as a strategy to socially integrate them in their workplaces and to increase their retention [30, 31].

Over the past few decades in Taiwan, the proportion of physicians employed by health care organizations has increased relative to independent physicians due to the general practice environment changing day-by-day and due to government policy (ex. the implementation of National Health Insurance in 1995). This is an issue that is quite similar throughout the world because of the change in general practice environment: many big hospitals, especially in medical centers, expend every effort and decision to focus on how to increase physicians’ welfare and quality of life, including economic incentives, hospital resources, and psychological aspects [32]. Therefore, we should attempt to understand why hospital physicians choose to shift their careers from employment at hospitals to become independent practitioners in clinics.

Most studies have explored hospital physician retention by measuring leaving intentions through employee surveys; however, this method might induce social desirability effects in the responses such that it deters accurately determining the reality of employee psychological status. Therefore, with independent physician practitioners who have already left hospitals as our subjects, this study is aimed at understanding their psychological work status regarding the hospital work environment and hospital resources at the time of their departure from their hospitals. The hope is that these findings can then provide future organizational managers or administrators with a better understanding of how to develop effective policies and to make better decisions that will improve physician retention.

## 2. Methods

### 2.1. Study sample and data collection

This study was designed as a cross-sectional survey using a multi-dimensional structured questionnaire to assess the level of job satisfaction of physicians that left their respective hospitals, including psychological work status and hospital resources. The 774 physicians included in our study were all from local medical clinics that were registered with the Department of Health, Executive Yuan, and located in Taichung city in the middle of Taiwan. A mail survey was sent to potential study participants during the period of January to March 2010, including a prepaid-postage envelope to return the completed paperwork. Follow-up calls were made to increase the response rate. In total, 353 clinic physicians out of 774 responded for a response rate of 45.6%.

### 2.2. Ethics

This study was approved by the Institutional Review Board of the China Medical University of Taichung.

**Table 1:** 

					Factor loadings†				
Question items		Mean	SD	Ranking ‡	(Factor 1) Job content	(Factor 2) Hospital environment	(Factor 3) Department environment	(Factor 4) Work motivation and retention	α
1.	Burdens of routine clinical work	3.47	0.83	10	0.75				0.87
2.	Burdens of routine administrative work	3.39	0.75	11	0.73				
3.	Job autonomy	3.57	0.90	7	0.72				
4.	Job development	3.61	0.92	5	0.60				
5.	Be respected on job	3.59	0.96	6	0.56				
6.	Doctor-patient relationship	3.89	0.75	1	0.66				
7.	Balance between work and family tasks	3.23	0.94	13	0.64				
8.	Opportunities to learn new skills and knowledge	3.56	0.90	8		0.74			0.75
9.	Opportunities to obtain specialty certificate	3.80	0.93	2		0.72			
10.	Opportunities to obtain teaching positions	2.93	1.07	16		0.61			
11.	Leadership in hospital executives	3.15	1.02	14		0.51			
12.	Leadership in working departments	3.30	0.96	12			0.60		0.83
13.	Peer cohesion in working departments	3.79	0.83	3			0.86		
14.	Overall working climate in working departments	3.71	0.83	4			0.86		
15.	Patient care coordination in working departments	3.51	0.76	9			0.50		
16.	Job equity	2.82	0.96	18				0.72	0.84
17.	Fringe benefits	2.71	0.86	19				0.79	
18.	Job security	3.07	0.99	15				0.66	
19.	Job prospects	2.92	0.96	17				0.57	

Note: 1. ^†^ Factor Analysis with the rotation method of Varimax with Kaiser Normalization. 2. ‡Higher numbering means less satisfied by the respondents and vice versa.

**Table 2:** 

					Factors loadings^†^		
Question items		Mean	SD	Ranking ‡	(Factor 5) Tangible resources	(Factor 6) Intangible resources	α
1.	Clinical workforce for clinical services	3.15	0.89	5	0.69		0.95
2.	Administrative workforce for clinical services	3.19	0.80	3	0.75		
3.	Financial resources for clinical services	3.02	0.87	8	0.80		
4.	Equipment resources for clinical services	3.15	0.90	5	0.74		
5.	Clinical workforce for teaching and research	2.90	0.91	10	0.81		
6.	Administrative workforce for teaching and research	2.91	0.89	9	0.86		
7.	Financial resources for teaching and research	2.79	0.90	12	0.87		
8.	Equipment resources for teaching and research	2.89	0.91	11	0.83		
9.	Patient service reputation among peers	3.52	0.76	1		0.86	0.92
10	Medical profession reputation among peers	3.47	0.80	2		0.89	
11.	Research profession reputation among peers	3.06	0.88	7		0.72	
12.	Medical teaching profession reputation among peers	3.18	0.89	4		0.79	

Note: 1. † Factor Analysis with the rotation method of Equamax with Kaiser Normalization. 2. ‡Higher numbering means less satisfied by the respondents and vice verse.

**Table 3:** 

Variables	Scales	Frequency	%	Mean	SD
**Personal characteristics**					
Age when leaving hospitals				37.16	6.37
Gender	Female	33	9.4		
	Male	318	90.1		
Surgery	No	301	85.3		
	Yes	48	13.6		
Internal medicine	No	234	66.3		
	Yes	115	32.6		
Obstetric/pediatrics	No	239	67.7		
	Yes	110	31.2		
Subspecialty	No	260	73.7		
	Yes	89	25.2		
Working hours per week					
Below 40 hours		66	18.7		
40-60 hours		179	50.7		
Above 60 hours		102	28.9		
Working years when leaving hospitals				7.08	4.94
Hospital characteristics					
Learning opportunity					
	Yes	180	51.0		
	No	125	35.4		
	Do not know	45	12.8		
Tenure opportunity					
	Yes	138	39.1		
	No	114	32.3		
	Do not know	100	28.3		
Promotion opportunity to attending physicians					
	Yes	275	77.9		
	No	39	11.1		
	Do not know	30	8.5		
Hospital ownership					
	Public	115	32.6		
	Private	154	43.6		
	Corporate	84	23.8		
Hospital location					
Outside the city		132	37.4		
Within the city		221	62.6		
Decade when leaving hospitals	1970s	108	30.6		
	1980s	163	46.2		
	1990s	54	15.3		
	2000s	20	5.7		

### 2.3. Study instruments

A multi-dimensional questionnaire was developed to assess the level of job satisfaction of physicians that left their respective hospitals with two dimensions: one, a 19-item part for examining psychological work status; and two, a 12-item part for examining hospital resources. These two dimensions were constructed from the proposed factors that might affect turnover decisions, including psychological, individual, organizational, environmental, and human resource-related factors [33-35]. The draft was first evaluated by several academic professionals and practitioners for content validity.

All of the items were measured on a 5-point Likert scale: strongly dissatisfied (1), dissatisfied (2), fair (3), satisfied (4), and strongly satisfied (5). Also, one additional scale, “not applicable”, was added for those respondents who had no experience with an item. Detailed information regarding the individual items are outlined in Table 1 and Table 2.

In addition, physicians’ demographics, working status, hospital characteristics, and the time of their leaving were collected in this study. Included data are gender, age at leaving, area of specialty, working hours per week, and number of years worked prior to leaving. The characteristics the physicians cited as related to leaving their respective hospitals included opportunities of learning, tenure, and promotion available to attending physicians, as well as hospital ownership and location. Also, the timing (*i.e.*, year) of physicians leaving was recorded. Detailed information about the individual items are outlined in Table 3.

### 2.4. Analytical techniques

The data were first analyzed descriptively by computing means and standard deviations for continuous variables and frequencies and percentages for categorical variables. Missing data were completed by using the mean variable for continuous variables of satisfaction evaluation. Other missing data for physician demographics were gathered by phone call or e-mail to ensure accuracy whenever possible.

Two factor analyses were performed for the 31 individual items of the psychological work status evaluation and the hospital resources evaluation, respectively. Four factor scores, “job content”, “hospital environment”, “department environment”, and “work motivation and retention”, were identified from the 19 items related to the psychological work status evaluation (see Table 1) by using factor analysis with the Rotation method of Varimax of Kaiser Normalization. Two factor scores, “tangible resources” and “intangible resources”, were identified from the 12 items related to the hospital resources evaluation (see Table 2) by using factor analysis with Rotation method of Equamax of Kaiser Normalization. Internal consistency measured as the Cronbach α value for the six factors just mentioned were 0.87, 0.75, 0.83, 0.84, 0.95 and 0.92, respectively. Other detailed descriptive analyses of these six factor scores are shown in Table 1, 2.

## 3. Results

### 3.1. Personal and contextual characteristics of resigned hospital physicians

Of the 353 respondents, 90.1% of the respondents were male and their ages ranged from 32 to 81 years (mean = 37.16 years). Respondents were comprised of Internal medicine physicians (32.6%), gynecologists and pediatricians (31.2%), surgeons (13.6%), and subspecialty physicians (25.2%), the latter including dermatologists, radiologists, psychiatrists, and so on. 50.7% of our respondents had to work 40-60 h per week and the average period they stayed in their respective hospitals was 7.08 years. 51.0% of the respondents said they had learning opportunities, 39.1% had tenure positions, and 77.9% had the opportunity to be an attending physician. Almost 60% (private 43.6% and corporate 23.8%) of hospitals involved were privatized hospitals and 62.6% hospitals were located in Taichung city. 46.2% of the doctors left their hospitals for independent practice in the 1980s.

Other descriptive analyses of personal and work characteristics of the physicians are shown in Table 3.

### 3.2. Psychological work status and hospital resources satisfaction of resigned hospital physicians: analysis of 31 individual items

Among the psychological work status evaluation’s 19 items, doctor-patient relationship (mean = 3.89) was ranked as the most satisfactory item, followed by opportunities to get a specialty certificate (mean = 3.80), then and peer cohesion in departments (mean = 3.79). Fringe benefits (mean = 2.71) was the least satisfactory item, while job equity (mean = 2.82) and job prospects (mean = 2.92) were also ranked within the bottom three.

Within the hospital resources evaluation’s 12 items, patient service reputation among peers (mean = 3.52) was ranked as the most satisfactory item, followed by medical profession reputation among peers (mean = 3.47) and administrative workforce for clinical services (mean = 3.19). Financial resources for teaching and research (mean = 2.79) was the least satisfactory item. We also found that the satisfaction score of every aspect in teaching and research was less than 3 (average score). Other descriptive analyses of hospital satisfaction and resources evaluation from physicians who have left hospitals are shown in Table 1 and Table 2.

### 3.3. Relationship between physician personal and contextual characteristics and the resigned hospital physicians’ satisfaction evaluation

Six factor scores extracted through factor analyses mentioned in the Methods section were used in the six multiple regression analyses as dependent variables, respectively, with demographics, hospital characteristics, and time of leaving as independent variables (see Table 4). This analysis revealed the following. Older physicians were less satisfied than younger physicians with regards to hospital environments. Male physicians were more satisfied with hospital tangible resources than female physicians. Internal medicine physicians were less satisfied with the intangible resources (*i.e.*, reputation) of their hospitals than non-internal medicine physicians. Gynecologists and pediatricians were more satisfied with hospital environments than non-gynecologists and pediatricians. The physicians that worked long hours per week were less satisfied with the job content at the hospitals they left. The physicians who had opportunities to learn advanced skills and were afforded knowledge development opportunities were more satisfied with their hospitals’ environments, tangible resources, and intangible resources (*i.e.*, reputation). In addition, physicians in private hospitals were more satisfied with their job content than those in public hospitals, but they were less satisfied with work motivation and retention and intangible resources (*i.e.*, reputation) than those in public hospitals. In addition, physicians that worked in hospitals located in Taichung city were less satisfied with tangible resources than those who worked in the hospitals outside Taichung city. All points mentioned above were shown to have a statistical significance of 0.05 level.

## 4. Discussion

This study explores the managerial implications from the perspective of both hospitals and individual physicians by evaluating the psychological status satisfaction and perceived hospital resources satisfaction of physicians that have left their respective hospitals to work in private practice. We found that older physicians were less satisfied as compared to younger physicians with regards to their hospital work environment. Male physicians were more satisfied with the tangible resources of hospitals than female physicians. Internal medicine physicians were less satisfied with the intangible resources (*i.e.*, reputation) of the hospitals they left than non-internal medicine physicians. Gynecologists and pediatricians were more satisfied with the work environment of hospitals they left than non-gynecologists and pediatricians. The physicians that worked long hours per week were less satisfied with the job content of the hospitals they left. The physicians who had had the opportunity to develop advanced skills and gain knowledge were more satisfied both in terms of tangible resources and intangible resources (*i.e.*, reputation). In addition, physicians in private hospitals were more satisfied with their job content than those in public hospitals, but they were less satisfied with work motivation and retention and intangible resources (*i.e.*, reputation) than those in public hospitals. Also, physicians who worked in hospitals located in Taichung city were less satisfied with the tangible resources than those who worked in hospitals outside Taichung city.

**Table 4:** 

Standardized Coefficients	(Factor 1) Job contents	(Factor 2) Hospital environment	(Factor 3) Department environment	(Factor 4) Work motivation and retention	(Factor 5) Tangible resources	(Factor 6) Intangible resources
**Personal characteristics**						
Age when leaving hospitals	0.01	-0.25*	0.07	0.17	-0.01	-0.14
Gender (default: female)	0.05	0.05	0.16	0.06	0.17*	0.03
Internal medicine (default: no)	0.05	0.03	0.01	0.16	0.27**	-0.20*
Obstetric/pediatrics (default: no)	0.06	0.21*	0.22	0.09	0.15	0.06
Subspecialty (default: no)	-0.04	0.08	0.13	0.10	0.15	-0.01
Working hours per week	-0.26**	0.02	0.03	0.10	0.02	-0.02
Working years before leaving hospitals	-0.04	0.14	-0.13	-0.14	-0.14	-0.15
**Hospital characteristics**						
Learning opportunity (default: no)	0.10	0.36***	-0.09	0.14	0.25**	0.21*
Tenure opportunity (default: no)	-0.03	0.04	0.03	0.11	0.13	-0.07
Promotion opportunity to attending physicians (default: no)	0.11	0.04	0.10	- 0.01	-0.01	0.08
Hospital ownership (default: public) Ownership: private	0.19*	-0.01	-0.05	-0.21*	-0.09	-0.18*
Ownership: corporate	-0.02	0.04	0.05	-0.04	-0.03	-0.01
Hospital location (default: outside city)						
Same city	-0.04	-0.05	-0.04	-0.11	-0.14*	-0.03
Decade when leaving hospitals	0.01	-0.14	0.12	0.01	-0.04	-0.06

*p < 0.05, **p < 0.01, ***p < 0.001

In this study, all of the 19 analyzed items of the psychological work status evaluation and the 12 analyzed items of hospital resources evaluation were ranked from a range of 2.79 to 3.89, and we found that doctor-patient relationship was the most satisfactory area and financial resources for teaching and research was the least satisfactory. Among the 31 items, only 8 analyzed items were of a below average score (average being 3), and they were opportunities to get teaching positions, job equity, fringe benefits, job prospects, clinical workforce, administrative workforce, financial resources, and equipment resources for teaching and research as perceived by hospital physicians. Previous studies have also shown that the fringe benefits of physicians was the least satisfactory item. Therefore, from the results mentioned above, hospital managers should pay more attention to improving work-related motivation, the retention of physicians, and perceived resources with regards to teaching and research.

In our study, we found that older physicians were less satisfied with the work environment of their hospitals, including opportunities to learn, obtaining specialty certificates, getting teaching positions, and executive leadership than younger physicians were. Previous studies revealed a slight statistical significance over leadership identification between older and younger physicians [34]. Traditionally, when physicians get older, they are already well experienced regarding their practice and knowledge, and the support hospitals provide perhaps no longer meets their expectations or they gradually developed their own values and opinions as they time passes. Based on our results, hospital managers should focus more on providing older physicians with a better hospital work environment and to make concerted efforts to understand how these older physicians really feel.

Also, it was found that male physicians were more satisfied with tangible resources than female physicians. Hospital resources such as essential medical facilities, sufficient space in examination rooms, and administrative staff supporting the hospital were associated with the job satisfaction of physicians, especially for male physicians [35]. McMurray *et al*. analyzed 5704 male and female physicians in their Physician Work Life Study, with a concern for sex differences [36]. They found that women were more likely than men to be dissatisfied, especially in the field of autonomy, relationships with community, pay, and, hospital resources. Therefore, it is the recommendation of this study that administrators should also take a closer look into how they can help the female physicians in their hospitals to help ensure greater retention.

According to our data, gynecologists and pediatricians were more satisfied with hospital work environments than the other specialties surveyed. Previous studies have revealed that the majority of gynecologists and pediatricians are satisfied with their career overall and believe in providing high quality care to patients with skill and knowledge [37]. They must stay at a hospital for a long time to deal with their unique professional work, such as, for example, gynecologists and delivering children. Therefore, supporting the easier obtainment of specialty certificates, new skills, and teaching positions as well as executive leadership should be focused on to make physicians feel more valued. We would recommend that managers look at the promotion process and certification of physicians, especially gynecologists and pediatricians. Additionally, we found that internal medicine physicians were more satisfied with the tangible resources of hospitals they left than non-internal medicine physicians. Internal medicine physicians might share a larger proportion of hospital budget and resources provided by hospitals. But because of our limited data, further studies will be needed to fully understand this particular phenomenon.

Furthermore, we found that physicians working long hours every week in hospitals they eventually left were less satisfied with the job content of their hospitals. The most frequently mentioned sources of job stress were increased workloads, paperwork, insufficient time to do justice to the job, and increased and inappropriate demands from patients [38]. The more work the physicians had to do, the less free time they had. Therefore, such a heavy burden might seriously impact their enthusiasm with regards to work. The more fatigue they experience, the less patience they have in their professional field. Moreover, physicians who have less patience might damage or compromise the delicate doctor-patient relationship and not be respected at their job. It is a vicious cycle in all occupations that the more hours employees put in, the less satisfaction they feel, and this has been proven in previous research [13, 39-40]. Thus, decreasing the amount of working hours weekly for physicians without affecting the profit of hospitals will be a challenging task for every manager who wishes to address the issue of physician retention.

The physicians who had opportunities for learning advanced skills and knowledge in their hospitals were more satisfied with the work environments of the hospitals they left as well as both the tangible and intangible resources (*i.e.*, reputation) of the hospitals. Determining a better way for physicians to get certifications and support from their respective hospitals would certainly be attractive and may help with retention. In addition, having a better reputation among peers in clinical work, research, and patient service would also naturally help to retain physicians more easily, especially those utilizing their advanced skills and knowledge. Previous studies on physicians’ satisfaction account for this phenomenon. For example, physicians employed by health care organizations who rated the quality of care they could provide as lower than ideal were less able to achieve their professional goals and were more likely to intend to leave their job [22]. Maybe managers can assist physicians with regards to obtaining professional certifications when establishing future hospital policies.

Research has already been published about the satisfaction of physicians and nurses [41-45]. However, studies that have focused on the differences between physicians’ working conditions and job satisfaction as they relate to the different types of hospitals is scarce [34]. Our study determined that physicians working at private hospitals were more satisfied with their job content than those in public hospitals; but, on the other hand, they were less satisfied with work motivation and retention and intangible resources (*i.e.*, reputation) than those in public hospitals. A separate study revealed that New Zealand radiologists’ working at public hospitals were less satisfied than at private hospitals in regards to work stress, burnout, and lower job satisfaction [46]. Another recent study revealed that teaching, research, and variety contribute more to academic satisfaction, whereas job autonomy, physicianpatient relationship, and coworkers contribute more to satisfaction for the physician in a private hospital [47]. The routine clinical and administrative work of privatized hospitals is more challenging and flexible relative to public hospitals; it is not immutable and frozen. A system of job responsibility is also implemented in a large portion of private hospitals. According to Herzberg’s two-factor theory, motivators, for example challenging work, recognition, and responsibility, result in the positive satisfaction of employee [48, 49]. Therefore, the burden of physicians may decrease and they could put more effort and energy into their doctor-patient relationships or to increase their level of job autonomy. Managers in public hospitals would do well to also focus on the job content of physicians.

It is worth highlighting that privatization leads to a change in ownership and aims to enhance an organization’s financial growth [50, 51]. It is move that is done to make hospitals more cost-effective and to augment financial growth [34]. Privatized hospital managers may overemphasize profit and thus may neglect employees’ fringe benefits, job equity, and this in turn may even affect job prospects and security, especially when the profit level of an organization is lower than the managers or executives expect. Herzberg argued that hygiene factors, for example, job security, salary, and fringe benefits, do not give positive satisfaction, though dissatisfaction results from their absence [48, 49]. This trend will result in a lower global budget for hospital clinical work, teaching, and research. The abovementioned reasons may account for why physicians working at private hospitals reported being less satisfied with work motivation and retention and intangible resources (*i.e.*, reputation). As regards intangible resources (*i.e.*, reputation), we have tried explain the results since related literature is not available. We explain it as follows. About two decades ago, the reputation of public hospitals was better than that of well-known private institutions in Taiwan. Many outstanding and professional physicians yearned to work for public hospitals due to the opportunity to do more research and also for the amount of teaching resources available. In other words, better employee welfare and benefits might very well attract more excellent physicians to join and stay with hospitals, especially executives in hospitals.

Finally, we found that physicians who worked in hospitals located in Taichung city were less satisfied with the tangible resources of the hospitals they left than those who worked in hospitals outside Taichung city. Previous studies about physician satisfaction that focused on hospitals’ locations are few and far between. A previous study showed that rural Minnesota physicians felt the least work stress about their feelings of clinical competence and interpersonal relations at work and anxieties about the future [52]. Urban and inner-city family physicians have reported seeing higher numbers of patients with complex disease profiles such as co-morbidities and emotional and mental health problems, compared with their suburban and rural colleagues [53]. It may be that the physicians working in Taichung city hospitals felt more stress with regards to teaching, research, and clinical service due to the greater number of hospitals as compared to outside Taichung city. To achieve a higher profit and to be more competitive, they had higher expectations about clinical and administrative workforce, equipment, and financial support. Perhaps they felt the tangible resources that hospitals provided them were still not enough. Relatively, most physicians working at hospitals outside Taichung city felt less stress and did not have high expectations about everything, which meant hospitals could meet their expectations. It can be said, then, that more financial and/or equipment support that related to teaching, research, and clinical practice would help hospital supervisors recruit more outstanding physicians in metropolitan areas.

## 5. Limitations

Certain limitations of this study should be pointed out. First, our data were collected from physicians who were involved with individual practice after leaving hospitals in Taichung city, Taiwan. Although it focused on all clinical physicians in Taichung city, the data revealed in our study may just show a local phenomenon based on the background of having a health care system supported by Taiwan’s National Health Insurance system, and thus may not be applicable to other countries. In the future, similar research into the issue of physician retention in hospitals should be extended to a wider area or should many counties in Taiwan. Such a broader scope will provide even more information for managers and supervisors in health care organizations.

Another limitation is that many respondents of this study were older and had already been involved with individual practice for more than 10 years after leaving their respective hospitals. As a result, recall bias may have occurred when answering the items on our questionnaire. We also provided an open question for the respondents to freely answer. A more flexible and dynamic evaluation would have provided our study team more information about the background of each doctor as it related to their leaving their institutions. For example, our respondents recommended that our questionnaire should pay more attention to satisfaction in different specialties or certain detailed items rather than the balance between work and family life.

The final limitation is that our study just focused on physicians that “left” their hospitals. Although this is also an advantage of this study, just focusing on psychological status and perceived resources satisfaction of physicians who had already left hospitals to practice privately may not differentiate the major or important determinants from those staying in hospitals. Perhaps exploring the relationship between major determinants and physicians that left their respective hospitals deserves future surveying.

## 6. Conclusion

Many studies have explored the level of satisfaction hospital physicians who were still working at a hospital from several different dimensions, such as a psychosocial perspective, a financial perspective, a general practice environment and even global satisfaction. However, few studies have focused on the satisfaction level of physicians who have left their hospital as opposed to the satisfaction level of physicians who have remained.

In this study of the psychological work status and the hospital resources evaluation of physicians who have left their respective hospitals, our conclusion is that there is still room for improvement with regards to work motivation and retention, financial and equipment support for teaching, research resources, and the opportunity to get teaching positions provided by health care organizations. All of these items scored at a below average level in our research. In addition to evaluating these two dimensions of physician satisfaction, we also examined the effects and relationship that physicians’ personal and professional characteristics had on them. It is our recommendation that hospital managers should pay attention to the real expectations or needs of retained physicians according to the results shown in our study and furthermore should adjust their managerial perspectives when establishing new human resources policies or decisions in order to hopefully improve the welfare and working conditions of hospitals for physicians in the near future.
